# Supplementary material for: ATR is essential for preservation of cell mechanics and nuclear integrity during interstitial migration
Source: Nat Commun. 2020 Sep 24;11:4828. doi: 10.1038/s41467-020-18580-9 (PMC7518249; doi:10.1038/s41467-020-18580-9)
Supplement: Supplementary file 1 — Supplementary Information [file 41467_2020_18580_MOESM1_ESM.pdf]

Supplementary Information for

ATR is essential for preservation of cell mechanics and nuclear integrity during interstitial migration

Gururaj Rao Kidiyoor, Qingsen Li, Giulia Bastianello[1] Christopher Bruhn, Irene Giovannetti, Adhil Mohamood, Galina V Beznoussenko, Alexandre Mironov, Matthew Raab, Matthieu Piel, Umberto Restuccia, Vittoria Matafora, Angela Bachi, Sara Barozzi, Dario Parazzoli, Emanuela Frittoli, Andrea Palamidessi, Tito Panciera, Stefano Piccolo, Giorgio Scita, Paolo Maiuri, Kristina M Havas, Zhongwei Zhou, Amit Kumar, Jiri Bartek, Zhao Qi Wang and Marco Foiani*

*Correspondence to MF: marco.foiani@ifom.eu

This file includes:

Supplementary Figure 1
Supplementary Figure 2
Supplementary Figure 3
Supplementary Figure 4
Supplementary Table 1

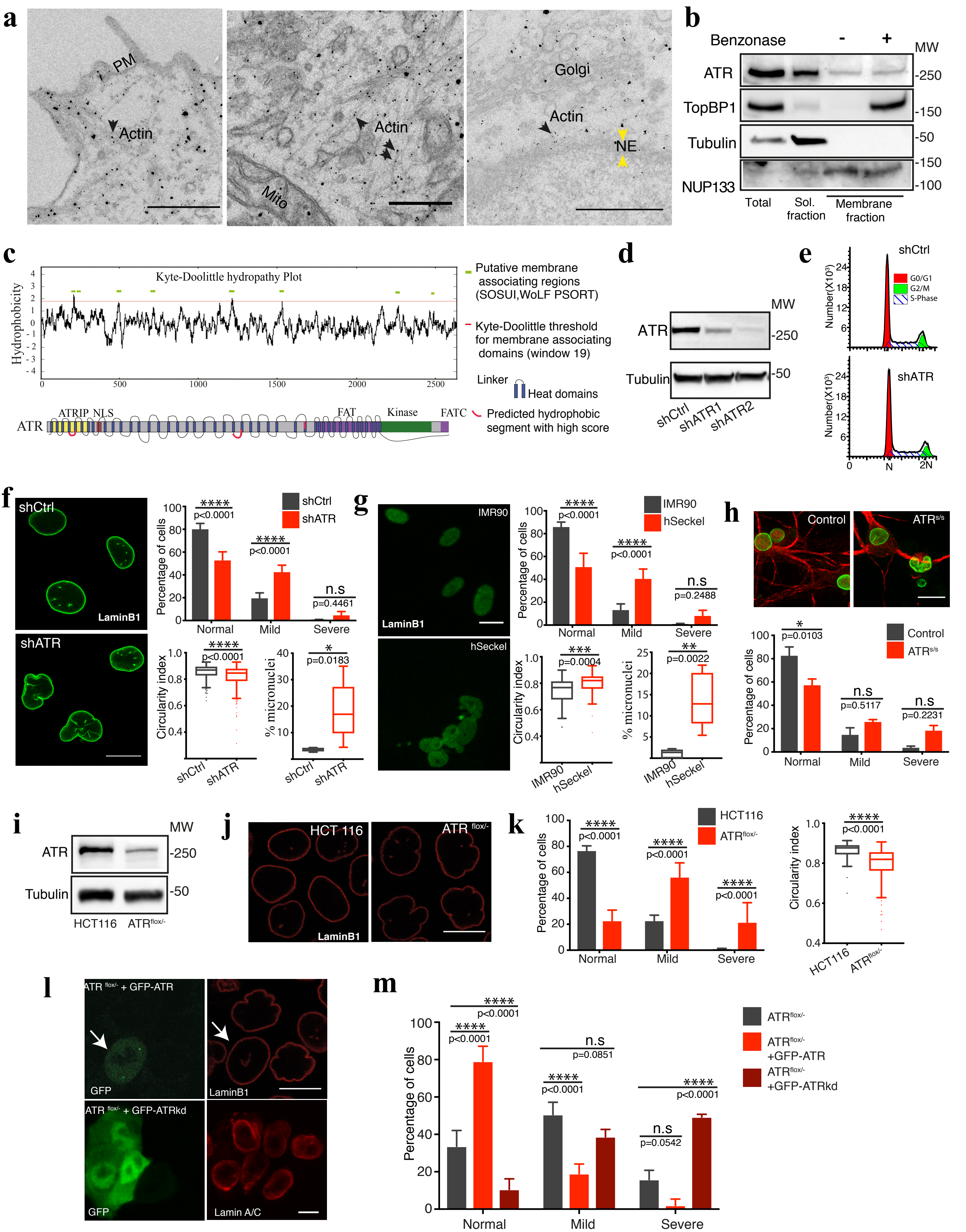
a
PM
Actin
Mito
Golgi
NE
b
Benzonase
-
+
MW
ATR
TopBP1
Tubulin
NUP133
-250
-150
-50
-150
-100
Total
Sol. fraction
Membrane fraction
c
Kyte-Doolittle hydropathy Plot
Hydrophobicity
Putative membrane associating regions (SOSUI,WoLF PSORT)
Kyte-Doolittle threshold for membrane associating domains (window 19)
Linker
Heat domains
Predicted hydrophobic segment with high score
ATR
ATRIP
NLS
FAT
Kinase
FATC
d
MW
ATR
Tubulin
-250
-50
shCtrl
shATR1
shATR2
e
shCtrl
shATR
G0/G1
G2/M
S-Phase
Number(X10³)
N
2N
f
shCtrl
shATR
LaminB1
Percentage of cells
Normal
Mild
Severe
****
p<0.0001
n.s
p=0.4461
Circularity index
% micronuclei
*
p=0.0183
g
IMR90
hSeckel
LaminB1
***
p=0.0004
**
p=0.0022
p=0.2488
h
Control
ATRs/s
*
p=0.0103
p=0.5117
p=0.2231
i
HCT116
ATRflox/-
j
HCT 116
ATR flox/-
LaminB1
k
HCT116
ATRflox/-
l
ATR flox/- + GFP-ATR
ATR flox/- + GFP-ATRkd
GFP
LaminB1
Lamin A/C
m
ATRflox/-
ATRflox/- +GFP-ATR
ATRflox/- +GFP-ATRkd
p=0.0851
p=0.0542

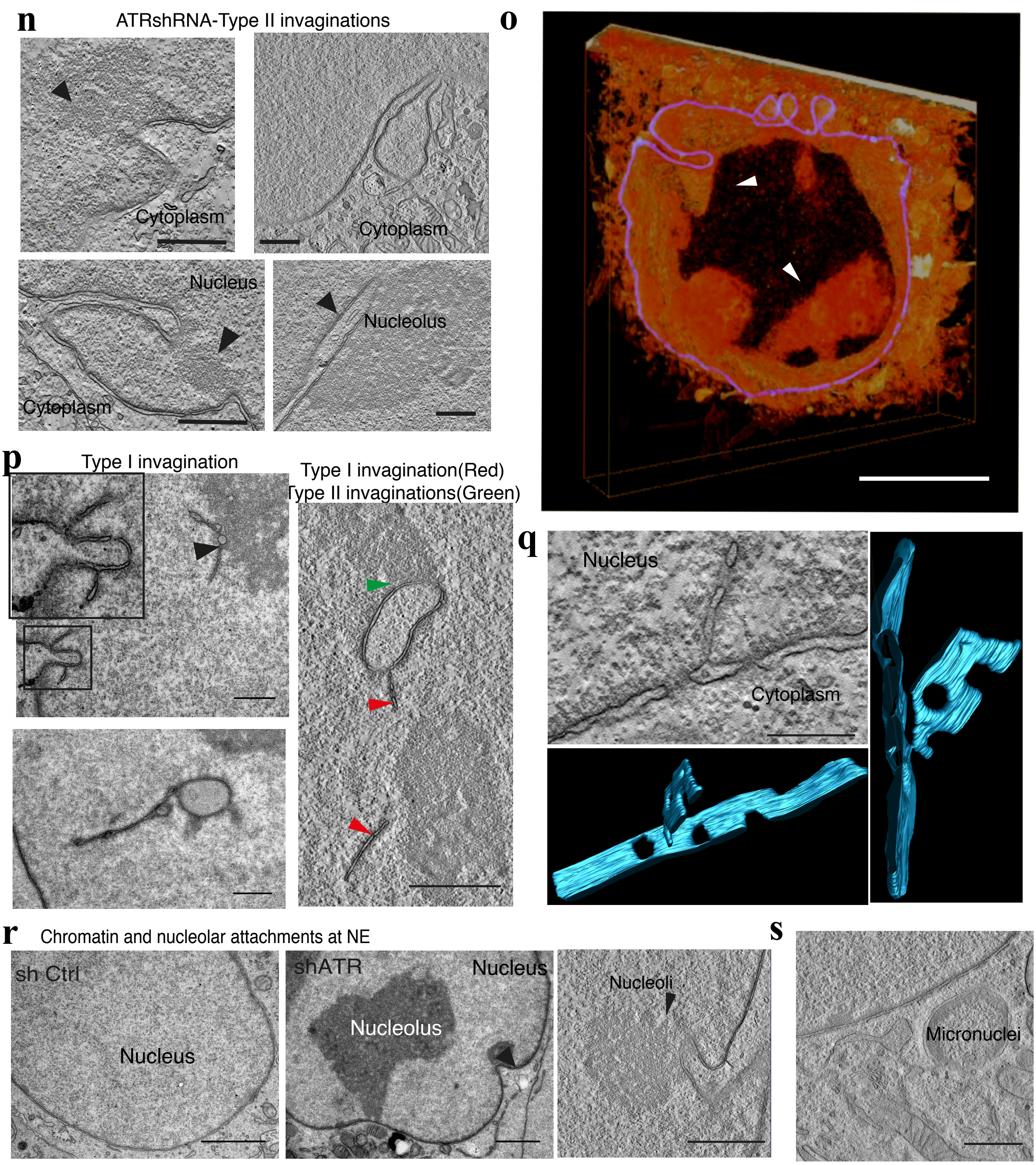
n
ATRshRNA-Type II invaginations
Cytoplasm
Cytoplasm
Nucleus
Cytoplasm
Nucleolus
o
p
Type I invagination
Type I invagination(Red)
Type II invaginations(Green)
q
Nucleus
Cytoplasm
r
Chromatin and nucleolar attachments at NE
sh Ctrl
Nucleus
shATR
Nucleus
Nucleolus
Nucleoli
s
Micronuclei

Supplementary Figure 1. ATR depletion alters nuclear morphology:
(a) ATR conjugated nano-gold particles are detected on the plasma membrane, nuclear membrane (yellow arrow) as well as attached to organelles and associated with actin fibers (black arrow). Scale bar is 1µm for right and left-side images and 500ηm for the central image. (b) Membrane fractionation of exponentially growing HeLa cells performed with Mem-PER™ Plus kit. Fractionation purity verified with anti-Tubulin (cytosol specific), anti-NUP133 (NE-specific) and anti-TopBP1 (chromatin binding) antibodies. Benzonase nuclease treatment (1 hour incubation) has no effect on the level of ATR in membrane fraction (c) Kyte-Doolittle hydropathy plot of ATR sequence, Red line indicates the threshold value for membrane association, green lines indicate putative membrane associating regions. (Lower panel) Overlapping these putative regions of membrane binding sequences into representative 3D structure of ATR. (d) immuno-blot of ATR knock-down efficiency by the shRNA used in this study. (e) Cytometric analysis of control and shATR cells show similar cell cycle profiles. Comparing nuclear morphology of (f) U2OS cells with shATR or control plasmids (n=333 and 299 cells for morphology and micronuclei analysis; 245 and 348 cells for circularity) and (g) human primary Seckel fibroblasts with normal human fibroblast as controls (n= 290 and 206 for morphology and micronuclei analysis; 81 and 80 for circularity measurements). Both shATR U2OS cells and human Seckel fibroblasts have higher number defective nuclear morphology, increased micronuclei and significantly altered nuclear circularity index (N=2 independent experiments). (h) Nuclear morphology defects in primary neurons from wild-type or seckel mice embryos (71 and 141 cells for wt and Seckel respectively; from 2 or 3 embryos each genotype). For (f,g,h) Scale bar is 20µm. (i-m) Recovery of nuclear defects by expressing ATR in $ATR^{flox/-}$ cells. (i) Western blot representing reduced ATR levels in HCT116 derived $ATR^{flox/-}$ cells; (j) immunofluorescence images (Scale bar is 10µm) and (k) quantifications showing defective nuclear morphology of $ATR^{flox/-}$cells compared to HCT116 cells (n= 449 and 426 cells, and for circularity n= 206 and 201 cells for HCT116 and $ATR^{flox/-}$; N=3 independent experiments). (l) Representative immunofluorescence images of nuclear morphology in presence $ATR^{flox/-}$ cells transfected with GFP-ATR, or GFP ATR-kd (Kinase dead form) and (m) quantifications of nuclear shape and circularity index (n= 317 ($ATR^{flox/-}$), 47 (GFP-ATR)) and 67 cells (KD-ATR-GFP)). (n-s) EM analysis of control nuclei and shATR nuclei. (n) Examples of type II nuclear invaginations (Scale bar is 1µm for left-side images and 650ηm for the right-side images). (o) 3D reconstruction of shATR nuclei section (arrowheads indicate NE invagination and NE attached nucleolus (Scale bar = 3µm). (p) Type II nuclear membrane invaginations observed in shATR nuclei (Scale bar = 1µm; inset ≈ 2X zoom). (q) 3D reconstruction of one of the type I nuclear invaginations (Scale bar = 300ηm) (r) Example images of nucleoli and condensed chromatin attached to the NE particularly at the invaginations (Scale bar = 2µm) and (s) micronuclei commonly observed in shATR nuclei (Scale bar = 1µm). Bar graphs presented as Mean +/- SEM and box plot whiskers and outliers plotted using Tukey method in prism-7 software. p-values calculated using Student t-test or Two-way or One-way ANOVA test with Tukey's multiple comparisons test. (****P<0.0001 ***P<0.001 **P<0.01 *P<0.05, ns – not significant).

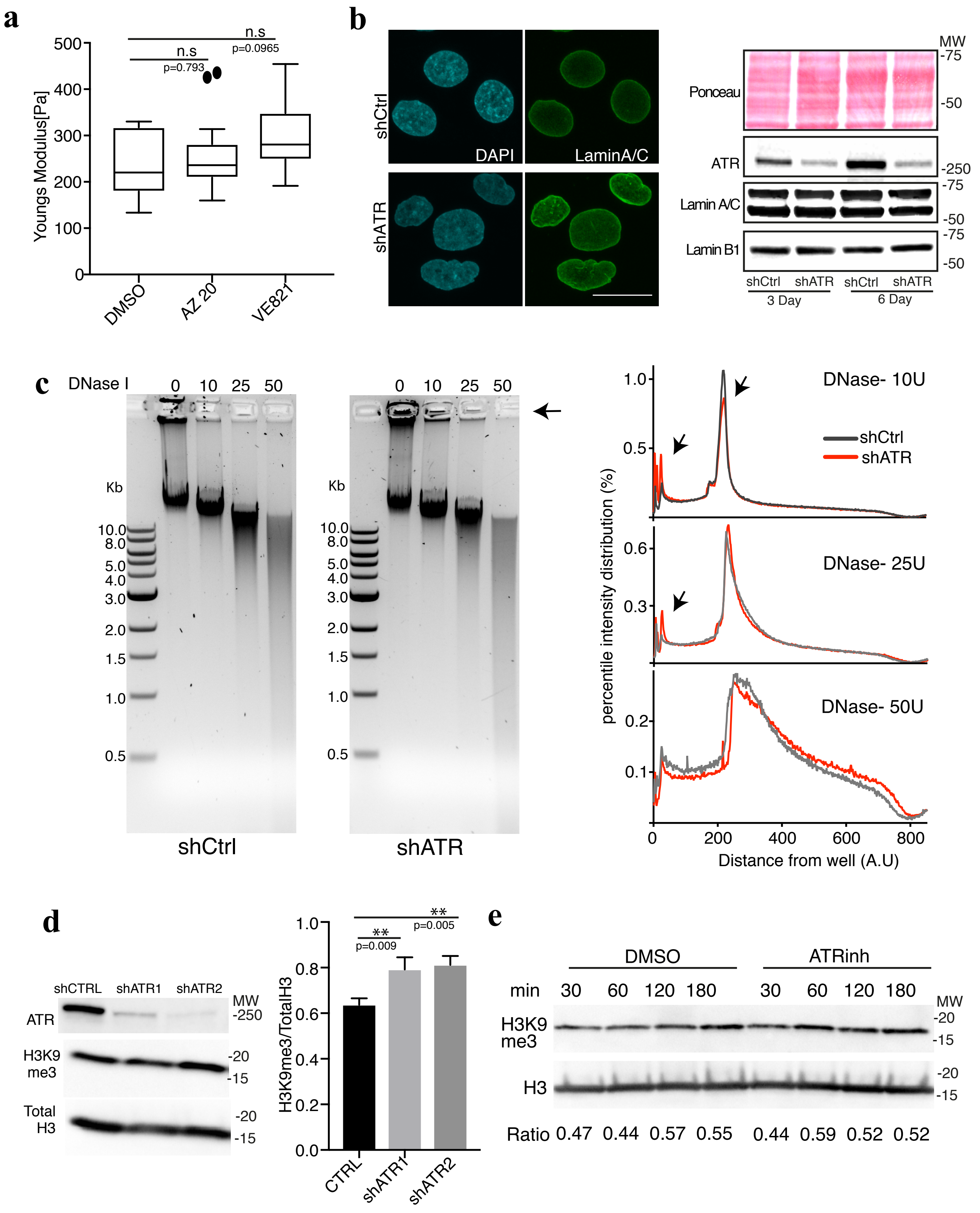
a
n.s
p=0.0965
n.s
p=0.793
Youngs Modulus[Pa]
500
400
300
200
100
0
DMSO
AZ 20
VE821
b
shCtrl
shATR
DAPI
LaminA/C
MW
-75
-50
-250
-75
-50
-75
-50
Ponceau
ATR
Lamin A/C
Lamin B1
shCtrl
shATR
shCtrl
shATR
3 Day
6 Day
c
DNase I
0
10
25
50
Kb
10.0
8.0
5.0
4.0
3.0
2.0
1.5
1.0
0.5
shCtrl
shATR
DNase- 10U
shCtrl
shATR
DNase- 25U
DNase- 50U
percentile intensity distribution (%)
Distance from well (A.U)
0
200
400
600
800
d
shCTRL
shATR1
shATR2
MW
ATR
-250
H3K9 me3
-20
-15
Total H3
-20
-15
**
p=0.005
**
p=0.009
H3K9me3/TotalH3
1.0
0.8
0.6
0.4
0.2
0.0
CTRL
shATR1
shATR2
e
DMSO
ATRinh
min
30
60
120
180
30
60
120
180
MW
H3K9 me3
-20
-15
H3
-20
-15
Ratio 0.47 0.44 0.57 0.55 0.44 0.59 0.52 0.52

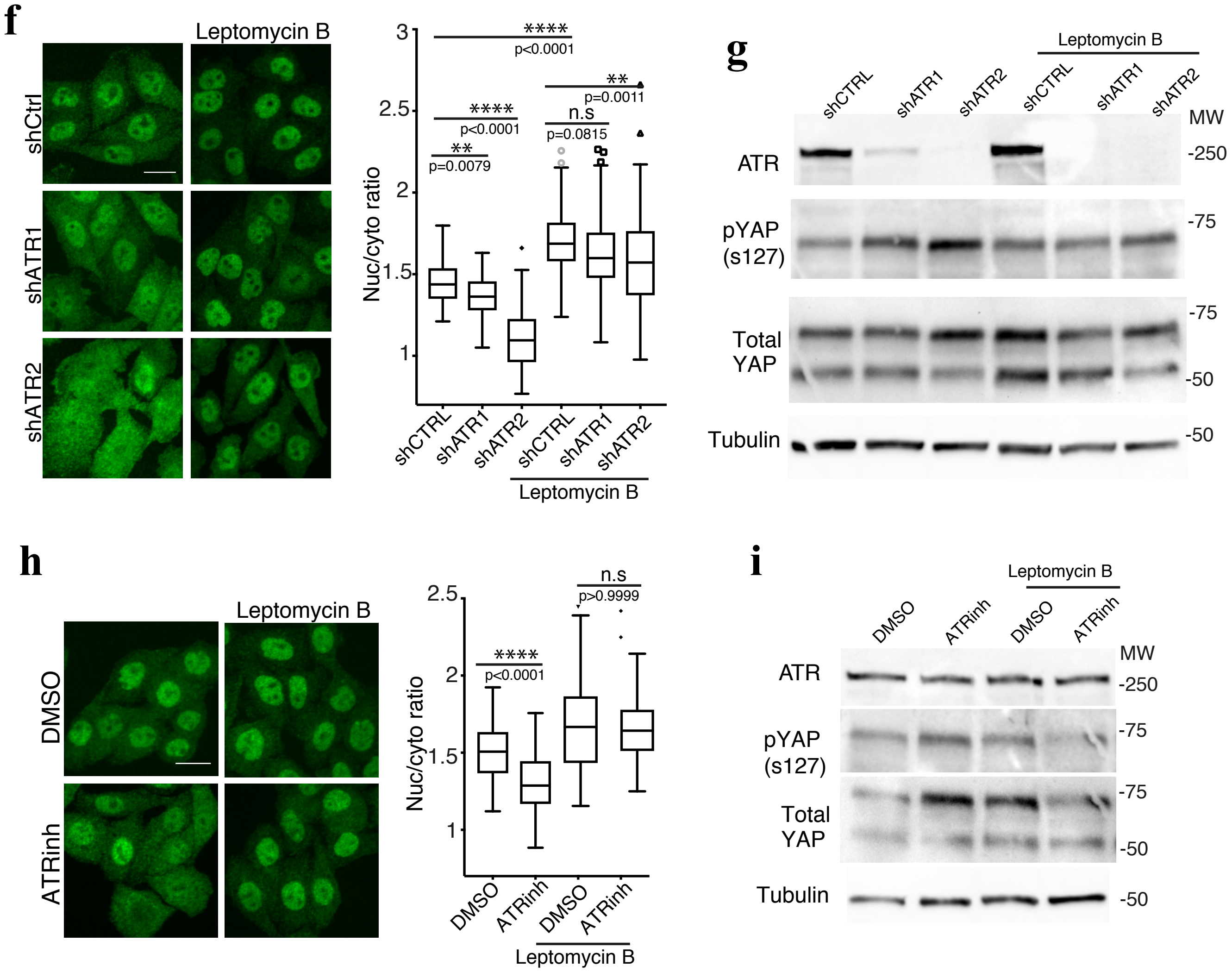
f
Leptomycin B
shCtrl
shATR1
shATR2
Nuc/cyto ratio
****
p<0.0001
**
p=0.0011
****
p<0.0001
n.s
p=0.0815
**
p=0.0079
shCTRL
shATR1
shATR2
shCTRL
shATR1
shATR2
Leptomycin B
g
Leptomycin B
shCTRL
shATR1
shATR2
shCTRL
shATR1
shATR2
MW
ATR
-250
pYAP
(s127)
-75
Total
YAP
-75
-50
Tubulin
-50
h
Leptomycin B
DMSO
ATRinh
Nuc/cyto ratio
****
p<0.0001
n.s
p>0.9999
DMSO
ATRinh
DMSO
ATRinh
Leptomycin B
i
Leptomycin B
DMSO
ATRinh
DMSO
ATRinh
MW
ATR
-250
pYAP
(s127)
-75
Total
YAP
-75
-50
Tubulin
-50

Supplementary Figure 2. Aberrant mechanical properties of the ATR-defective nucleus: (a) AFM measurements (Elastic modulus) of cellular stiffness in presence of ATR inhibitors for 4 hour (n= 64, 58, 49 measurements from 17, 14 and 11 cells respectively; two independents experiments performed and data from one measurements is shown). (b) Lamin A/C show no significant changes in the cellular localization (IF analysis) and protein expression levels (western-blot analysis). (c) Gel image and graph of relative intensity of DNAse sensitivity assay: Isolated nuclei form control and shATR cells are treated with varying concentrations of DNAse I for 15 minutes. Arrowheads indicate the regions of interest (N=2, data from one experiment is presented). (d-e) Western blot and quantifications showing alterations in level of H3K9 trimethylation in: (d) control and shATR cells or in (e) cells acutely treated with ATR inhibitor (VE821). (f-i) YAP localization in presence of leptomycin B (10ng/ml); (f) Western blot analysis of ser-127-phosphorylated YAP in ATR depleted cells with or without leptomycin B (3hr) (g) Immunofluorescence images and quantifications of nuclear to cytoplasmic YAP signal ratio (n= 113, 110, 113 cells for untreated; n=102,102,109 cells for lept B treatment). (h) Western blot analysis of ser-127 phosphorylated YAP in presence of ATR inhibitor (VE821) in cells pretreated with Leptomycin B. (i) Immunofluorescent images and quantifications of nuclear to cytoplasmic YAP signal ratio of same treatment (n=107 (DMSO), 103 (ATRinh). 125 (DMSO-LeptB), 101 cells (ATRinh+LeptB)). Scale bar = 20μm. Bar graphs presented as Mean +/- SEM and box plot whiskers and outliers plotted using Tukey method in prism-7 software. p-values calculated using One-way ANOVA test with Dunnett's, Tukey's or Bonferroni's multiple comparisons test (****P<0.0001 ***P<0.001 **P<0.01 *P<0.05, ns – not significant).

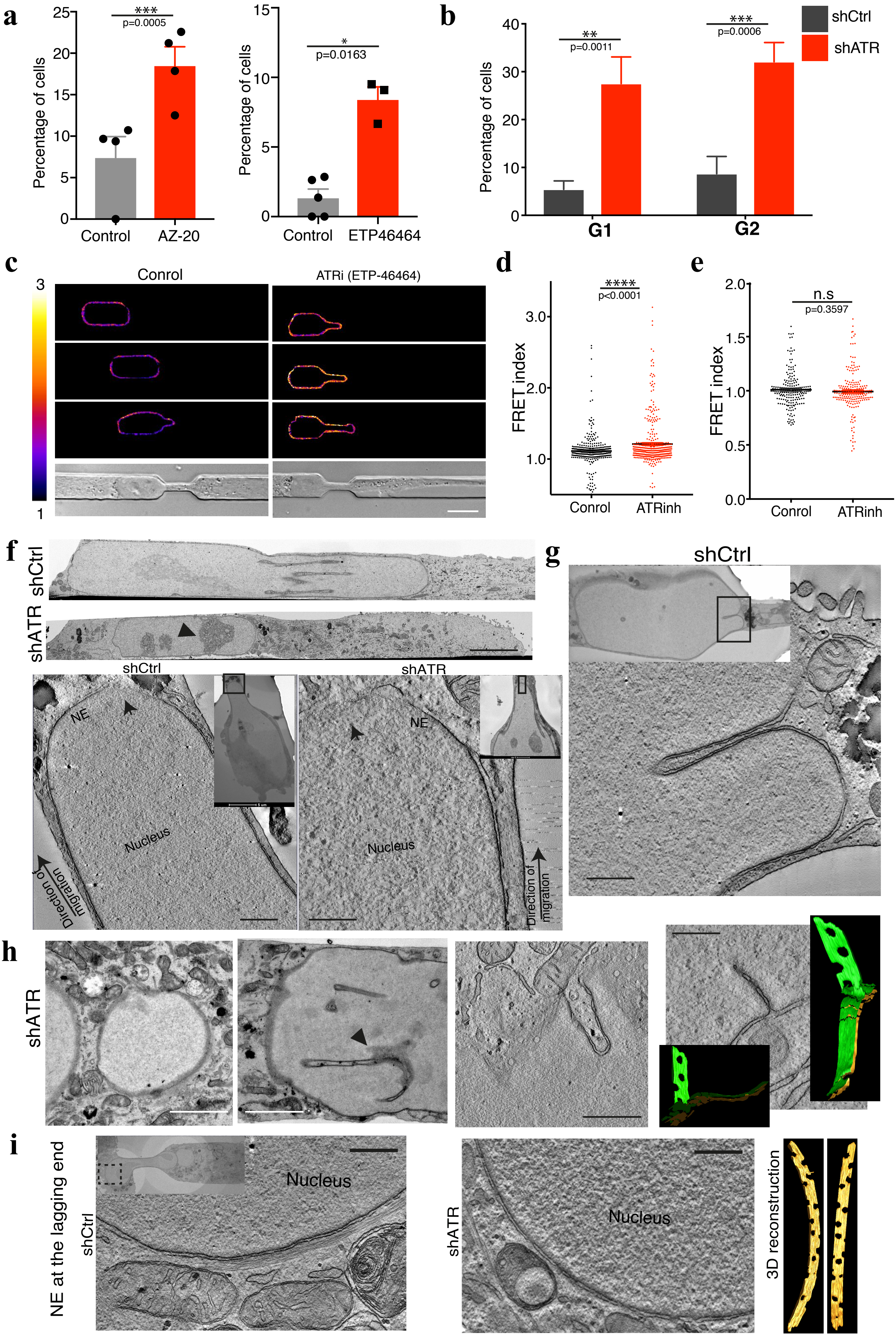
a
Percentage of cells
***
p=0.0005
Control
AZ-20
*
p=0.0163
Control
ETP46464
b
Percentage of cells
**
p=0.0011
***
p=0.0006
shCtrl
shATR
G1
G2
c
Conrol
ATRi (ETP-46464)
d
FRET index
****
p<0.0001
Conrol
ATRinh
e
FRET index
n.s
p=0.3597
Conrol
ATRinh
f
shCtrl
shATR
NE
Nucleus
Direction of migration
g
shCtrl
h
shATR
i
NE at the lagging end
shCtrl
Nucleus
shATR
Nucleus
3D reconstruction

Supplementary Figure 3. Analysis of ATR-defective nuclei during interstitial migration: (a) Percentage cell death in H2B-mCherry labeled HeLa cells, passing through the constriction in presence of ATR inhibitors (n=145 and 74 for ETP464 and n=117 and 124 cells for AZ-20); data pooled from 1 to 3 independent experiments). (b) Quantification of cell death in U2OS cells stably expressing the cell cycle markers (FUCCI) passing through the channel (n=154 and 212 cells for shCtrl and shATR1 respectively)). (c-e) Nuclear tension is differentially distributed in the leading and lagging edge of a cell migrating along constriction (c) Representative images of FRET signal distribution in control cells and ATR depleted cells during migration in channel with constriction. Scale bar = 20µm. (d) Nesprin-2 tension sensor measured FRET signal of cells treated with DMSO or ATR inhibitor (ETP46464) (n= 332 and 424 measurements). (e) FRET signal quantifications from headless control of Nesprin-2 tension sensor, of cells treated with DMSO or ATR inhibitor (ETP46464) (n= 196 and 200 measurements). (f) EM images of control and shATR HeLa cells in constriction (FIB SEM). Arrows indicate damaged NE at the leading edge (Scale bar = 5µm for top panels and 1µm for lower panels). (g) Nucleus of control HeLa cell entering constriction with invagination (routine 200-ηm EM section; Scale bar = 2µm for first two panels and 1µm for the remaining). (h) Examples of NE abnormalities observed in shATR HeLa nuclei in constrictions including micronuclei, invaginations, increased nucleoli or chromatin association with NE (Scale bar = 1µm) (i) EM images of posterior section of control and shATR NE with intact morphology with no damage (Scale bar = 1µm). Statistical analysis performed with two-tailed student t-test or two-way ANOVA test with Sidak's multiple comparisons test (* if $0.01<p<0.05$; ** if $0.005<p<0.01$; *** if $0.001<p<0.005$; **** if $p<0.001$).

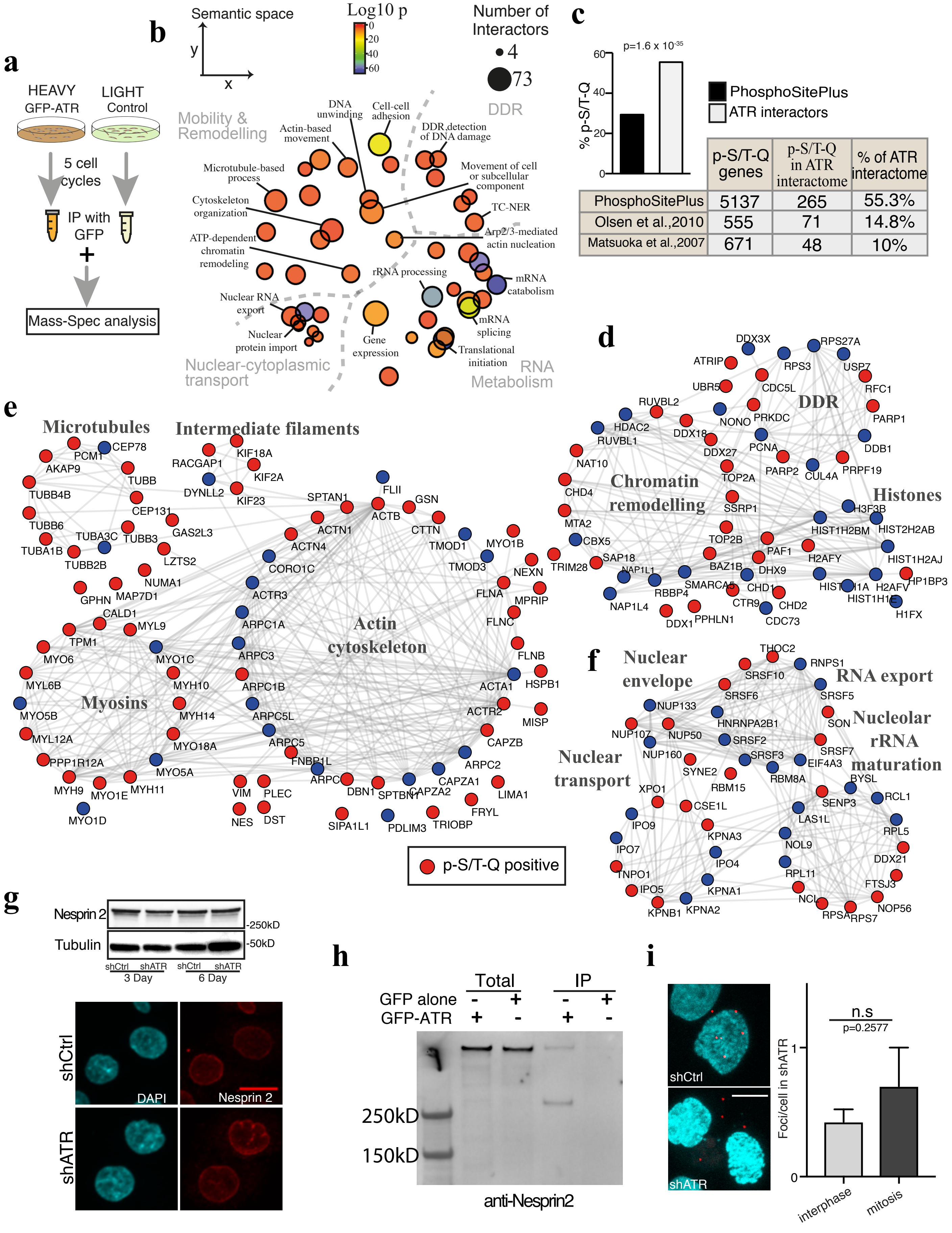

	p-S/T-Q genes	p-S/T-Q in ATR interactome	% of ATR interactome
PhosphoSitePlus	5137	265	55.3%
Olsen et al.,2010	555	71	14.8%
Matsuoka et al.,2007	671	48	10%

Supplementary Figure 4. Analysis of ATR interactome:

(a) Scheme of SILAC experiment design. (b) Gene ontology (GO) enrichment analysis of ATR interactors for biological processes. Significantly enriched GO terms are plotted in REVIGO semantic space, with similar GO terms remaining close together in space. The size of the dots represents the number of proteins and color gradient represents the log10 of p-value. Boundaries subcategorizing the GO terms were drawn manually. (c) ATR interactome is compared with (Phosphoprotein database) PhosphositePlus and other published datasets to score for potential ATR targets. (d-f) STRING interaction analysis of manually curated sub-categories of ATR interactors namely nuclear, nuclear envelope associated and cytoplasmic. In red are the positively scored proteins for phosphorylation at p-S/T-Q in databases (d) Nuclear proteins cluster in histones, DDR and chromatin remodeling processes. (f) Nuclear envelope proteins formed networks of NE components, RNA export, other nuclear transport and nucleolar RNA maturation. (e) Cytoskeletal partners of ATR included actin cytoskeleton components, myosin, and microtubule and intermediate filament proteins. (g) Nesprin-2 shows no significant change in the protein levels (western-blot analysis) and cellular localization (IF analysis; scale 20µm) in shATR HeLa cells compared to controls. (h) Immuno-blot of nesprin-2 from figure 6b with molecular weights included. (i) Images and quantifications of PLA foci in ATR-depleted cells (n=33 and 10 cells; two tailed student t-test p= 0.2577).

Supplementary Table 1. Primers used in this study

Construction of GFP-ATR-KD	
GFPATR1	ACACACGGATCCATGCGGTGACGTCGAGGAG
GFPATR2	ACACACATTTAAATAGACAGGTTCAATATTTCTATAAGC
GFPATR3	CTCGGCGCGGGTCTTGTAGTTGCCGTCG
ATR1	ATACCTACACTTCCATCAATTCTGGG
ATR2	GAGACTCTGCATCTTTTCTTAAGCAC
CMV1	CATGACCTTATGGGACTTTCCTACTTGG
CMV2	AATTAATACGACTCACTATAGGGAGACCC
Primers used for genotyping Atr-iKO mice	
ATR10	CTATTTTTTGTTGCTGGTTTTG
ATR15	CTTCTAATCTTC-CTCCAGAATTGTAAAAGG
Cre1	CGGTCGATGCAACGAGTGATG
Cre2	CCAGA-GACGGAAATCCATCGC
