# Supplementary material for: ATR is essential for preservation of cell mechanics and nuclear integrity during interstitial migration
Source: Nat Commun. 2020 Sep 24;11:4828. doi: 10.1038/s41467-020-18580-9 (PMC7518249; doi:10.1038/s41467-020-18580-9)
Supplement: Supplementary file 3 — Description of Additional Supplementary Files [file 41467_2020_18580_MOESM3_ESM.pdf]

Description of Additional Supplementary Files

File Name: Supplementary Data 1
Description: ATR interactors list : Manually curated list of ATR interactors from 3 proteomic experiments.

File Name: Supplementary Movie 1
Description: 3D reconstruction of shATR HeLa nucleus using Electron Microscopy.

File Name: Supplementary Movie 2
Description: Interstitial migration of control and shATR HeLa cells labeled with H2BmCherry. Scale bar =20μm
