# Supplementary material for: ATR is essential for preservation of cell mechanics and nuclear integrity during interstitial migration
Source: Nat Commun. 2020 Sep 24;11:4828. doi: 10.1038/s41467-020-18580-9 (PMC7518249; doi:10.1038/s41467-020-18580-9)
Supplement: Supplementary file 7 — Reporting Summary [file 41467_2020_18580_MOESM7_ESM.pdf]

nature research

Corresponding author(s): Marco Foiani
Last updated by author(s): Aug 3, 2020

Reporting Summary

Nature Research wishes to improve the reproducibility of the work that we publish. This form provides structure for consistency and transparency in reporting. For further information on Nature Research policies, seeAuthors & Referees and theEditorial Policy Checklist .

Statistics

For all statistical analyses, confirm that the following items are present in the figure legend, table legend, main text, or Methods section.

n/a	Confirmed	
☐	☒	The exact sample size (*n*) for each experimental group/condition, given as a discrete number and unit of measurement
☐	☒	A statement on whether measurements were taken from distinct samples or whether the same sample was measured repeatedly
☐	☒	The statistical test(s) used AND whether they are one- or two-sided<br>*Only common tests should be described solely by name; describe more complex techniques in the Methods section.*
☒	☐	A description of all covariates tested
☐	☒	A description of any assumptions or corrections, such as tests of normality and adjustment for multiple comparisons
☐	☒	A full description of the statistical parameters including central tendency (e.g. means) or other basic estimates (e.g. regression coefficient) AND variation (e.g. standard deviation) or associated estimates of uncertainty (e.g. confidence intervals)
☐	☒	For null hypothesis testing, the test statistic (e.g. *F*, *t*, *r*) with confidence intervals, effect sizes, degrees of freedom and *P* value noted<br>*Give P values as exact values whenever suitable.*
☒	☐	For Bayesian analysis, information on the choice of priors and Markov chain Monte Carlo settings
☒	☐	For hierarchical and complex designs, identification of the appropriate level for tests and full reporting of outcomes
☒	☐	Estimates of effect sizes (e.g. Cohen's *d*, Pearson's *r*), indicating how they were calculated

Our web collection on statistics for biologists contains articles on many of the points above.

Software and code

Policy information about availability of computer code

Data collection	All immunofluorescent data and live cell imaging are acquired using custom software provided by the microscope manufacturer, namely-Velocity software PerkinElmer (version 6.3), OLYMPUS cellSens Dimension (v1.18), Leica Application Suite X software (v. 3.5.2.18963), DeltaVision Elite imaging system, image Lab v5.0 (Biorad), Mascot (v. 2.3.02), : Lipidview workstation (version 1.3 beta, AB SCIEX, USA), PicoQuant software (SymPho Time 64, ver. 2.4)
Data analysis	Image J (1.49o), Graphpad Prism5 and 7, Microsoft excel (2011), Cytoscape (version 3.5.1), QuickTime Pro 7.5 (apple), 3DMOD (4.0.11), image Lab v5.0 (Biorad), Scaffold (v. 4.3.4). Revigo (http://revigo.irb.hr/), DAVID (https://david.ncifcrf.gov/), R-studio (1.0.153), STRING 11.0(https://string-db.org/), Atlas3D software (FIBICS) Amira 5.3.1(FEI, Thermo Fisher Scientific, Eindhoven. the Netherlands), Metaboanalyst 4.0 (https://www.metaboanalyst.ca/MetaboAnalyst/faces/home.xhtml).

For manuscripts utilizing custom algorithms or software that are central to the research but not yet described in published literature, software must be made available to editors/reviewers. We strongly encourage code deposition in a community repository (e.g. GitHub). See the Nature Research guidelines for submitting code & software for further information.

Data

Policy information about availability of data

All manuscripts must include a data availability statement. This statement should provide the following information, where applicable:

- Accession codes, unique identifiers, or web links for publicly available datasets
- A list of figures that have associated raw data
- A description of any restrictions on data availability

All data are available in main text, supplementary materials and/or in source data file. Uncropped western-blots, details of statistical analysis are included in source data file. The mass spectrometry proteomics data have been deposited to the ProteomeXchange Consortium via the PRIDE partner repository with the dataset identifier PXD020622.

Field-specific reporting

Please select the one below that is the best fit for your research. If you are not sure, read the appropriate sections before making your selection.

☒ Life sciences ☐ Behavioural & social sciences ☐ Ecological, evolutionary & environmental sciences

For a reference copy of the document with all sections, see nature.com/documents/nr-reporting-summary-flat.pdf

Life sciences study design

All studies must disclose on these points even when the disclosure is negative.

Sample size	Sufficient sample s for statistical significance, at least two and often more were undertaken. The sample size is indicated for each experiment.
Data exclusions	No data exclusions were necessary for this study.
Replication	Data was successfully replicated in at least 2 independent experiments and can be reproduced. details of the replication are included in figure legends of Individual experiment.
Randomization	Cells were analyzed from randomly assigned fields for quantifications
Blinding	Blinding was not possible for our experiments due to experimental design and low sample size often only two (control and ATR-depletion). However, all imaging were acquired and quantified using the least biased approach possible.

Reporting for specific materials, systems and methods

We require information from authors about some types of materials, experimental systems and methods used in many studies. Here, indicate whether each material, system or method listed is relevant to your study. If you are not sure if a list item applies to your research, read the appropriate section before selecting a response.

Materials & experimental systems

n/a	Involved in the study	
☐	☒	Antibodies
☐	☒	Eukaryotic cell lines
☒	☐	Palaeontology
☐	☒	Animals and other organisms
☒	☐	Human research participants
☒	☐	Clinical data

Methods

n/a	Involved in the study	
☒	☐	ChIP-seq
☒	☐	Flow cytometry
☒	☐	MRI-based neuroimaging

Antibodies

Antibodies used

1. ATR Cell signal 2790
2. TopBP1 Abcam ab2402
3. Nup133 SantaCruz sc-27392
4. Tubulin Sigma T5168
5. Lamin B1 Abcam ab16048
6. Lamin A/C Santa cruz sc-7292
7. Nesprin 2 Thermoscientific MA5-18075
8. Histone H3(tri-methyl K9) Abcam ab8898
9. total Histone H3 Abcam ab1791
10. Total YAP(63.7) Santacruz sc-101199
11. Phospho YAP (Ser127) Cell signal 4911S

Secondary antibodies:
Polyclonal Donkey anti-mouse AlexaFluor-488 AB_2340846 (Jackson ImmunoResearch)
Polyclonal Donkey anti-mouse AlexaFluor-594 AB_2340854 (Jackson ImmunoResearch)
Polyclonal Donkey anti-mouse AlexaFluor-Cy3 AB_2340813 (Jackson ImmunoResearch)
Polyclonal Donkey anti-rabbit AlexaFluor-488 AB_2313584 (Jackson ImmunoResearch)
Polyclonal Donkey anti-rabbit AlexaFluor-594 AB_2340621 (Jackson ImmunoResearch)
Polyclonal Donkey anti-rabbit AlexaFluor-Cy3 AB_2307443 (Jackson ImmunoResearch)

Validation	TopBP1 Abcam ab2402<br>Lamin B1 Abcam ab16048<br>Histone H3(tri-methyl K9) Abcam ab8898<br>total Histone H3 Abcam ab1791<br>https://www.abcam.com/primary-antibodies/a-guide-to-antibody-validation<br><br>ATR Cell signal 2790<br>Phospho YAP (Ser127) Cell signal 4911S<br>https://www.cellsignal.com/contents/our-approach-antibody-validation-principles/antibody-validation-for-immunofluorescence/ourapproach-validation-if<br><br>Lamin A/C Santa cruz sc-7292<br>Nup133 SantaCruz sc-27392<br>Total YAP(63.7) Santacruz sc-101199<br>https://www.scbt.com/resources/protocols/immunofluorescence-cell-staining<br><br>Nesprin 2 Thermoscientific MA5-18075<br>https://www.thermofisher.com/content/dam/LifeTech/Documents/PDFs/PG1705-PJT2509-COL05292-RO-Ab-Validation-Wiley-MiniBook-Americas-Final.pdf<br><br>Tubulin Sigma T5168<br>https://www.sigmaaldrich.com/technical-documents/articles/biology/antibody-standard-validation.html

Eukaryotic cell lines

Policy information about cell lines

Cell line source(s)	U2OS cells, HeLa cells, HCT116 and ATRflox/- cells were from The American Type Culture Collection (ATCC), maintained by IFOM cell culture facility. Human primary Seckel fibroblasts (GM18366) and IMR90 were from Coriell Cell Repository.
Authentication	none of the cell lines used were authencated
Mycoplasma contamination	negative. Mycoplasma tests are regularly conducted to ensure contamination free cells.
Commonly misidentified lines<br>(See ICLAC register)	none

Animals and other organisms

Policy information about studies involving animals; ARRIVE guidelines recommended for reporting animal research

Laboratory animals	1. B6/CBAF1 mice - Electroporation was performed in pregnant (E14.5) mother.<br>2. Atr-CER (129/Sv and C57BL/6 mixed background)- pregnant (E13.5) were used for cell collection from embryos.<br>All animal experiments were approved by Thüringen Landesamt für Verbraucherschutz(TLV), Germany. The pregnant mothers were between 3~8months of age.<br>3. NOD SCID gamma (male with age 6-8 weeks) -All animal experiments were approved by the OPBA (Organisms for the well-being of the animal) of IFOM and Cogentech. All experiments complied with Italian national guidelines and legislation for animal experimentation. All mice were bred and maintained under specific pathogen-free conditions in our animal facilities at Cogentech Consortium at the FIRC Institute of Molecular Oncology Foundation and at the European Institute of Oncology in Milan, under the authorization from the Italian Ministry of Health (Autorizzazione N° 604-2016).<br><br>All animals were housing at a temperature 22 ° C ± 2, humidity 30-70%, 12 h / 12 h dark/light cycle.
Wild animals	None
Field-collected samples	none
Ethics oversight	All animal experiments were approved by the Italian ministry and in accordance with Italian/German national and international laws and policies. Mice were bred and housed under pathogen-free conditions in our animal facilities at Cogentech Consortium at the FIRC Institute of Molecular Oncology Foundation.<br>All animal experiments were approved by Thüringen Landesamt für Verbraucherschutz(TLV), Germany.or by the OPBA (Organisms for the well-being of the animal) of IFOM and Cogentech, under the authorization from the Italian Ministry of Health (Autorizzazione N° 604-2016).

Note that full information on the approval of the study protocol must also be provided in the manuscript.
